# Philadelphia County Medical Society

**Published:** 1852-04

**Authors:** 


### PHILADELPHIA COUNTY MEDICAL SOCIETY.

*Extra Meeting, March 9th,* 1852.

### DISCUSSION ON ANÆSTHESIA.

(Reported for the Medical Examiner.)

Dr. Condie, in opening the discussion, said: It would have been better, perhaps, had some one who is an advocate for the employment of Anæsthetics under all the circumstances in which they have been recommended, to have opened the discussion this evening, but as I have been requested to commence, I feel no hesitation in announcing myself as one entirely opposed to their employment when this is done for no other object than merely to prevent or assuage pain.

Pain I admit to be an evil, often of very considerable magnitude, to relieve which is unquestionably the duty of the physician, in every instance in which it can be done without endangering the patient's life, or incurring the risk of augmenting the violence and duration of any disease under which the patient may at the time labor. But to prevent or soothe pain is not certainly the leading object of the physician's office—on the contrary, his chief aim should be to protect the organism from every lesion by which its functions may be impaired, or its life destroyed. To do this he is often warranted in inflicting pain, but he has no right to jeopard the integrity or life of the organism, merely for the sake of relieving or preventing temporary suffering. The propriety of the employment of anæsthetics for no other object than to produce insensibility to pain, must be based upon their entire and invariable safety. The advocates of these agents maintain that when they are used with proper precaution, and their effects are carefully watched, they are perfectly safe. The rules, however, for their safe employment have never been given with any degree of clearness and precision.

Whether all the cases have been made public, in which the induction of insensibility by means of the recently introduced anæsthetic agents has been productive of injurious or fatal results; cannot be ascertained, but a sufficient number are upon record to prove that these agents are not as invariably safe, as was at first supposed. It is now admitted by some of the advocates for their employment, that we cannot always predict the effects that may result from their administration—that we are not, as yet, able to determine, before hand, the existence of those constitutions or peculiar states of the system in which the production of anæsthesia is liable to prove injurious. Now upon this are based my objections to the use of anæsthetics, when given merely to induce insensibility to pain. In surgical operations, and during parturition, it is not maintained, by any one, so far as I am aware, that the induction of anæsthesia increases in either case the safety of the patient—it is admitted, I believe on all hands, that the only benefit that results is simply relief from pain. This is unquestionably, in most instances, a boon not to be refused to our patient when it can be safely granted; but I cannot admit the right of the surgeon or obstetrician to resort to an anæsthetic to prevent the pain of an operation, or that of parturition, in any instance, until he shall be able to say, positively, that no injury will be liable to result from its employment. I can conceive of cases where it would be injudicious to cause insensibility to pain, even though it might be done without the slightest risk—cases where the sufferings of the patient afford important indications to the surgeon or accoucheur, upon the absence of which indications he is led into errors that may eventuate in much suffering, or even the loss of life.

It has been asked whether, if we could be put in possession of the statistics of the deaths from opium, the destruction of life resulting from its use would not far exceed that which has been caused by any of the modern anæsthetic agents, and whether with this fact staring him in the face, the practitioner would be warranted in abstaining from its use as a remedial agent in the cases which call for its administration. It cannot he denied that much mischief—a great destruction of life, even, has been occasioned by the *careless* and *improper* employment of opium, in common with every other active article upon the lists of the materia medica, and I for one, certainly do not admit that, on this account, its use as a therapeutic agent is to be denounced. But is any practitioner prepared to defend the administration of opium for the production of anæsthesia in surgical operations and during parturition? If so, then I admit that the argument in favor of the use of anæsthetics in surgery and obstetrics, drawn from the occasional injurious effects of opium, has some force; but not otherwise.

For the purpose of arresting disease, of guarding the organs of his patient from dangerous lesions, and thus saving him from death, from protracted suffering, or from decrepitude, it is the duty of the physician to make use of every therapeutic means which experience has shown to be generally beneficial in similar cases—nay, where the disease is violent and the danger of a fatal result imminent, he is warranted in the resort to remedies, which, from their well-known properties, would appear to be adapted to fulfil promptly the well marked indications in the case under treatment, even though we are aware that the use of those remedies is attended with danger—that in place of a curative, they may possibly produce a destructive influence. But in cases that he has every reason to believe will terminate favorably without the administration of any doubtful or dangerous remedy, is there any propriety in the practitioner resorting to one of this character, merely to relieve some prominent and distressing symptom? I should certainly say there is not. I know not what others would feel, if by a remedy given merely to cause insensibility to pain, in any case of which pain is known not to enhance the danger, the death of the patient were to be produced; but I trust that, with me, every conscientious physician would acknowledge that the plea of a humane intention to save his patient from suffering, is not a sufficient excuse for the fatal effects of his indiscretion.

As therapeutic means, the modern anæsthetic agents, even when administered by inhalation, may prove advantageous, I have no doubt, in many violent and dangerous diseases. Their topical application to external parts, when these are the seat of severe neuralgic pains, has been shown, in a large number of instances, to give prompt and effectual relief.

Some of the advocates for the use of anæsthetics in surgery and obstetrics, admit that danger may result from the inhalation of chloroform, but insist that such is not the case when pure sulphuric ether is inhaled—in no instance, they assert, has the slightest injury resulted, when this latter has been employed for the production of insensibility to pain. This sweeping assertion is, I confess, a startling one, when we reflect upon the immense number of cases in which the sulphuric ether is reported to have been employed, and the careless manner in which it must at first have been administered, and then consider the powerful impression it exerts upon the brain and nervous system when inhaled, as it has so often been, to a sufficient extent to induce complete anæsthesia. I admit, at the same time, that, if there has been no keeping back of the fatal cases—there is scarcely any thing upon record to disprove the innocuous character that has been ascribed to sulphuric ether as an anæsthetic.

It has been recently reported, but I will not vouch for the accuracy of the statement, that although the production of insensibility by the inhalation of anæsthetic agents may not produce immediately any injurious results, that the impression made by these agents upon the brain has subsequently resulted in a derangement or impairment of the intellectual faculties. I can easily conceive that this might result from their frequent use, at short intervals, but have my doubts as to its having ever occurred from a single employment of them, excepting, perhaps, it may have been in a case, where the brain was already strongly predisposed to disease or functional disturbance, and then, we should presume that the result would be immediate or nearly so.

Dr. Turnbull, though opposed to the indiscriminate use of anæsthetic medicines, expressed himself as greatly in favor of sulphuric ether. He considers it the safest and most reliable of this class of agents, and stated that he had been able to find but one case recorded of death caused by its use. This is narrated in the number of Ranking's Abstract, for June 1848, p. 191, and is as follows:—

"On the 10th of July, a man æt. 55, of robust constitution, was etherised for the removal of a tumor; after inhaling two or three minutes, considerable agitation was observed in the face and limbs; during five minutes more the inhalation was continued, and complete insensibility induced. The first incision was performed, when the dark color of the patient's countenance attracted the operator's attention, and the man almost immediately expired. On dissection, the viscera exhaled a powerful odor of ether, the blood was viscid, and the lungs were deeply congested."—*Journ. des Connais. Med.-Chirurg.*

Three other cases are recorded; an investigation of which, however, will show that death was erroneously attributed to the ether employed.

The number of deaths from chloroform, amounted to 18; a difference very disparaging to the latter.

The mischief, in most of the fatal cases recorded, he was firmly convinced, resulted not so much from the article per se, as the abuse, or incautious use of it.

Now ether and chloroform are generally administered by means of a handkerchief, and consequently, as the medicine is impermeable to the atmosphere, there is not a due admixture of oxygen with the ethereal vapor; hence, the oxygenation of the blood is impeded and dangerous, and occasionally, as we have seen, fatal results ensue.

In administering ether, we endeavor to bring the system rapidly under its influence, by applying to the mouth of the patient a sponge well charged with the anæsthetic. In the use of chloroform, more caution is required; fifteen drops should be exhibited and repeated every minute or two, until the desired effect is obtained.

Dr. T. regarded the use of anæsthetic agents as invaluable in many diseases, in which pain is a prominent and distressing symptom; as in colic, asthma, neuralgia, &c.

Dr. Parrish remarked, that in the absence of so many of the advocates of anæsthetic agents, he felt called upon to say something in their favor, in reply to the remarks of the former speaker.

It so happened, said Dr. P., that on the first announcement of the discovery of the power of ether to destroy pain, I was one of those who received the intelligence with gladness. It came to us in the autumn of 1846, on the authority of Drs. Warren and Hayward, two of the oldest and most eminent Surgeons of New England, attested by their colleagues, at the Massachusetts General Hospital, and by other reliable witnesses of its effects. The evidence was too strong to be resisted, and I distinctly remember the effect which the reading of Dr. Bigelow's first paper on the subject produced on my mind. It sent a thrill of delight and gratitude through my frame as I read that an agent had really been discovered, which had the power of promptly and effectually destroying the sense of pain, even under the most severe inflictions of the knife.

For, whatever may be said here of theuses of pain, and however lightly we may esteem its dangers, it is still a dreaded evil. Before it the stoutest heart quails, the strongest man becomes helpless as a child, and the bravest man reveals that he is as weak and frail as the rest of mortals. Under its severe attacks all alike succumb and cry out for relief. But, besides the mere interruption to ease and comfort induced by pain, it is often a positive evil, and may even prove a cause of death.

It aggravates disease by disturbing the balance of the nervous system, and preventing that repose so necessary to the recuperative process, while, if it be intense and long continued, it may exhaust the nervous power, and extinguish life itself.

Whatever tends, therefore, to annul pain is not a mere luxury, but a positive remedial agent of great power and utility. Hence, I cannot but regard the discovery of ether as a great boon conferred upon mankind, and although not naturally as ardent and sanguine in my temperament as some of my friends, I was not yet cold enough to receive the tidings of its introduction with calmness or indifference.

I confess, however, that a shade came over the brightness of the first view, when I reflected that an agent possessing such remarkable powers, might, on more extensive trials, prove so eminently dangerous to life as to preclude its employment. I fancied some poor patient stretched on the operating table about to be subjected to a safe and simple operation, suddenly overpowered by its use beyond the point of recovery; and the image of a scene so frightful rose up before me as a thick cloud on the sky of hope.

I became from the first an anxious, and I trust, an unprejudiced inquirer into the merits of this discovery; and holding, at that time, the position of Reporter on the improvements in Surgery, for the College of Physicians, I made the subject of anæsthetic agents a special topic of investigation. After the lapse of a year, the evidence, pro and con, was carefully sifted—the medical journals of this country and of Europe were critically examined, and notwithstanding many of these had at first taken strong ground against ether, and were by no means backward in publishing anything which was deemed unfavorable to it, an overwhelming mass of testimony to its utility and safety was collected. I found that, although the article had been used in thousands of cases, from the simple operation of extracting a tooth, to the most extensive and dangerous operations in Surgery; although its employment had been almost indiscriminate, and in many instances it had been recklessly given by non-professional persons—that but eight or ten cases of death had been attributed even by its opponents to its use—and that not one of these occurred under its immediate effects, the event generally occurring days, and even weeks afterwards, and under circumstances which rendered it very questionable whether the ether had any agency in the result. Since that time, which was in the year 1847, I have been an attentive observer of the progress of this matter. I have examined carefully for fatal results, and have not yet found a case reported in which death could be fairly and unequivocally attributed to the action of ether.

The case mentioned by Dr. Turnbull this evening, may be one of that character, and similar ones may have been reported, but if so, they have escaped my observation.

As it relates to chloroform, their can be no doubt that eighteen deaths have occurred after the use of this article in surgical operations, and some of them under circumstances where all needful caution was observed in its administration. These may have been owing to some idiosyncrasy in the individuals submitted to its action, or to a peculiar mental or nervous condition existing at the time. Fright from the apprehension of an impending operation, or faintness from mental inquietude, while the patient was sitting upright, in connection with the strong and sudden sedative influence of the chloroform, may have extinguished the vital spark in some of these cases. But whatever the cause, they stand against the reputation of chloroform as a safe article, although they are not, I think sufficient, indiscriminately to condemn it.

It is worthy of remark, that all the fatal cases from chloroform have occurred in surgical practice; and so far as I have examimed into them they have generally been before the infliction of pain. When given in cases of labor, where the pains are violent, and for the purpose of counteracting them, it seems to be more safe; and I am not aware of any deaths being reported by obstetric physicians, although it has been extensively employed in their department.

The objection made here to anæsthetics, on the ground of the dangers to be apprehended from their *injudicious* employment, will lie against all the most potent and useful articles of the Materia Medica. Shall morphia be condemned because by the mistake of a physician or apothecary, a solution of a drachm to an ounce of water in a teaspoonful dose will kill, in a case where a grain to the ounce was intended? Shall laudanum be set aside, because it is taken in large doses for the purpose of self-destruction? Shall wine and brandy be stricken from our list of remedies, because thousands are annually swept into their graves from their habitual use? The holding up of this argument by the opponents of anæsthetic agents, shows the shifts to which they must resort to support their position, for they are possessed of too much intelligence and good sense to use it under any other circumstances. Considering the great power of these agents, and the rapidity with which they act, it is indeed remarkable that so few fatal cases have occurred. Let it be remembered that ether has now been in use for over five years, and chloroform for over four years, and that they have been extensively employed in all parts of the civilized world; that every large Hospital in Europe and America, except our own Pennsylvania Hospital in Philadelphia, uses one or the other of them habitually before great operations; that private practitioners employ them daily in surgical and obstetric practice, and yet with all this vast experience of their use, their most determined opponents can with difficulty make out a single case of death directly traceable to ether, and but eighteen attributable in any way to chloroform; and I say that no article in the Materia Medica is established upon a firmer basis. It is in vain to interpose theoretical objections, or imagined fears, against such an array of facts; and because a man may have once committed himself by a hasty opinion, it is useless to continue the opposition against such evidence.

As it relates to my personal experience of the use of anæsthetics, I may remark, that as to chloroform I have rarely used it, regarding ether as equally effectual and much more safe; and while that is attainable it is unnecessary to resort to the other.

If ether was not at hand, and the case was urgent, I should not hesitate however to employ chloroform, with that caution which the power of the article renders it necessary to observe. I have many times administered ether, and although for several years past my attention has been mainly devoted to general practice, almost to the exclusion of obstetrics, and of large operations in surgery, yet I have often been associated with others in the management of important surgical cases, and have had frequent opportunities of observing the effect of ether in preventing the pain of operations. Quite recently, Dr. Pancoast and myself were consulted by a lady from New Jersey with a large tumor on the side of the neck, involving a lobe of the parotid gland, which we deemed it proper to extirpate. The operation was skilfully performed by Dr. Pancoast while the patient was under the influence of ether. In this case the article acted most happily. The patient sat upright in a chair during the operation, with her head leaning on my shoulder; she was sufficiently conscious to reply to questions, while the feeling of pain was completely annulled. After the Dr. had dissected off the skin from the tumor and was dragging it from its bed, in order more completely to detach it from the surrounding parts, the patient apparently lying in a tranquil slumber, I inquired of her how she felt? when she replied in the tone of a person overcome with drowsiness, "very comfortable!" After the completion of the operation, she stated that the last thing she remembered was the sensation of the knife cutting through the skin, as though a line were being drawn over it by a dull instrument; and when the tumor was shown to her, she could scarcely believe that it had been removed. There was in this case no moaning, or stertorous breathing, or restlessness while under the influence of the ether, and she came out of it cheerful and self-possessed. Union by the first intention took place in the wound, and she had a rapid convalescence.

I have been concerned with Dr. Pancoast still more recently, in two cases of cancerous mammæ, in which, after presenting to the patients and their friends the doubts hanging over the result of an operation, they have decided to submit to it, if they could take ether.

In both cases the glands of the axilla were involved, and it was necessary to prolong the incision into the axilla, and to pick out the glands with the fingers. This was done without the patients being conscious of it, although in both of them there was some degree of restlessness and apparent distress during the operation. One lady experienced under the ether a pleasurable excitement, and awoke while the dressings were being applied, in an excellent humor, and with that tendency to loquacity which is often observed after the use of alcoholic stimuli. It would be easy to multiply cases of this description, but I will not detain the Society longer. I may say, however, that I have used ether to a considerable extent as a therapeutic agent, in nervous diseases, and in some cases where there was an intolerance of the preparations of opium, and yet where it was very desirable to induce sleep. Such are cases of nervous or sick headache, of neuralgia, various spasmodic affections, &c. In these cases I have seen the inhalation of small quantities of ether given at short intervals, and not carried far enough to induce complete insensibility, produce the most happy effects. About a tablespoonful poured upon a sponge, previously moistened with hot water, and taken in the hands of the patient, will often tranquillize the system, and favor repose, where anodynes fail, or produce but a partial effect; nor have I ever seen any unpleasant effect produced by this mode of administration. I am glad that my friend Dr. Condie does not carry his objections to the use of anæsthetics so far as to condemn them altogether in the treatment of disease, and that he is willing to admit that they may prove advantageous in many violent cases; though I cannot see how with these views he can be so decidedly against them in surgical operations and in the practice of obstetrics.

I know of no subject which can more properly claim the attention of the Society than this; and it is to be hoped that the members will express their views freely upon it.

We in Philadelphia are, I fear, regarded by our brethren in other parts, as too indifferent to the value of this discovery, and from the fact of some of our leading men taking strong ground against it at the start, many may have been disposed to shut their eyes to the true position of the case, and have not given to it a free and unprejudiced inquiry.

I trust, however, that though slow and cautious in forming an opinion, the profession in Philadelphia will be found right on this question, and that no one will allow his preconceived views to stand in the way of its fair and candid examination.

Dr. Darrach, said: That upon all new medical topics, diversity of opinion may be very properly and necessarily expected to exist; some will entertain views in favor and others in opposition; nevertheless, in the midst and by means of it, the profession is quietly and slowly forming its axiom.

In respect to anæsthetic agents, as two views, strongly in contrast with each other, have been eloquently presented, it may not be without interest to give the subject a triangular form, by presenting a third view.

The agencies in question, more especially chloroform, are dangerous. Chloroform has destroyed life, and that in a most unexpected, sudden, and shocking manner, and had it been discovered in the more depraved ages of the world, it would doubtless have subserved tragical designs. And, although we now have no such fears, yet independently of the sad effects of presumptuous ignorance, there is still danger from its use; and that when under the ordinary watchfulness of the physician. In an obstetrical case, for example, chloroform was administered with ordinary prudence, or rather say, within the limits of *ordinary imprudence*, and the patient became quickly insensible, unconcious, pulseless and cool, and all this continued long enough to agonize the attending physician with the fear of horrid and fatal consequences—his tormenting imagination quickly depicting the lifeless body of a woman in labor, and the consequent grief, inquest and trial.

Nevertheles, anæsthetics, as the nullifiers of pain, mark a new epoch in medicine—a blessing. The dentist, surgeon, obstetrician, and the general practitioner, are all called upon to use it. It removes the sting of disease, operations, and the cursed pain of child-birth. Man's punishment is to obtain his food by the sweat of his brow—hard labor! and woman's to have pains in child-birth. But the law is satisfied, and now, since man is blessed through christianity with *labor* saving machinery, that he may no longer toil, woman in child-birth must not judicially and cruelly be denied chloroform, her pain-saving boon in labor.

Hear the testimony of a lady on this point. "I inhaled the chloroform by applying the mouth of the phial to one nostril, whilst for safety I breathed the atmosphere with the other; and without disturbing consciousness or pulse, the tremor, physical and mental, from fear of the approaching labor, which agitated my whole frame, gave place to a most delightful soothing; the subsequent grasping pains were subdued, and the ordinarily severe forcing pains at the close were rendered altogether tolerable. I now, she adds, look back with gratitude at the whole course of the labor as divested of its accustomed dread and pain."

In view of such testimony it becomes an important question, whether the physician, whose proper position is that of a servant to remove not only disease but suffering, is at liberty to withhold that, which is not the property of the physician, but the boon for suffering humanity—a gift of christianity—especially so to woman in her agony.

These pain-saving agents have also enlarged the domain of surgery, greatly diminished the mortality of its operations, and are making it more popular and applicable. Operations are by them rendered practicable, which otherwise could not be so from their duration, frightful character and extreme and protracted painfulness. They prevent the *deadly shock* from acute and protracted pain which the surgeon otherwise inflicts; how many from the *shock* of the operation have died! By the formation of the sentiment that the capital operations are painless, they will make the *early* resort to them more customary. Here is a most invaluable benefit. The surgeon is no longer the butcher-knifeman! His sternness gives place to benignity, and his relieved patient awakes to the joy that without suffering he is restored to health. As the office pupil of the late Dr. Physick, and subsequently for years an observer of European surgery, especially that by Dupuytren at the Hotel Dieu, it is my painful testimony that capital operations, without anæsthetic agents, have heavy mortality; the surgeon, like the soldier, may boast of his nerve and firmness, but he must weep over the slain. How true is this in regard to the mortality from lithotomy. The cause is evident; none, not even the most intelligent, far less the uneducated, have submitted to this fearful and hazardous operation—a choice between inevitable and possible death—until the bladder and contiguous parts have become congested, irritated and disorganized by innumerable fits of stone, the calculus augmented in size, and mortification from the operation hazarded. But now by chloroform and early application, this dreadful operation has become painless and successful.

In respect to dentistry, the extraction of not only a tooth, but of many teeth at a sitting, has become painless and safe.

As to its modus operandi, Dr. Simpson and others have considered it that of asphyxia, a state of direct and immediate pulselessness. But a minute observation of its phenomena will distinguish them from those of all the recognized forms of asphyxia. These relate directly rather to blood; those in question to nerve; and may therefore be better explained by the Hall doctrine of the nervous arc.

The animal surfaces more especially the dermel and mucous, are esodic nervous surfaces. They have the spreading terminals of the esodic nerve of sensation. These terminals being directly impressed by chloroform, their function is suspended without otherwise affecting the constitution. Continue the application, and the sensorium becomes affected, and thereby consciousness is suspended; still further continue the application, and the heart's action is arrested. Thus we may properly deduce three stages in the action of chloroform; 1st, loss of sensation of the esodic surface, partial or general; 2d, loss of sensation, and consciousness; 3d, loss of sensation, consciousness, and pulsation, which last being continued becomes death.

The first of these stages is entirely safe; the second may also be considered without positive danger; but the third is fearfully hazardous. These stages may well be represented by the three breakers on the sea shore; to bathe in the first is safe, in the second approaches the verge of danger, and in the third is to be the undertow and death.

The intelligent dentist may then safely use chloroform if he *limits* its effect to the said primary stage, beyond which he need not extend it. A lady urgent for its benefit in the extraction of some teeth, breathed the chloroform a few seconds without the least affecting consciousness. After which the gums were immediately cut, she then declared that she felt the instrument moving about the crown of the teeth, but that it produced no pain. Again, in like manner, the agent was administered, and then three large teeth were successively extracted. She testified that she felt all the manipulations, but that they were each and all of them painless.

Dr. Emerson observed that in regard to the agencies by which unconsciousness to pain was induced, there were some phenomena connected with the nervous system and its mysterious functions, which he thought had not been generally recognized, or if noticed, not regarded with the attention which they deserved. He referred to the power of suspending sensibility exhibited by many persons subjected to pain.

The mesmerists, he said, claimed this power as one of the results of their manipulations, and many cases are related where cancerous breasts have been cut out, and other most formidable operations performed on patients previously mesmerized, who declared they were free from pain whilst under the hand of the Surgeon. He would not dispute the facts in such cases, further than to deny the necessity of any such fallacious agent as animal magnetism to induce the condition. The power evoked, through mesmerism, was doubtless exerted by the minds of very impressible subjects. He had often observed that certain persons seemed endowed with a capacity, on some occasions, to throw the ordinary functions of the nervous system "out of gear," to use a familiar term of machinists. Dr. Emerson related a few cases illustrating the subject. In one of these, he was assisting the late Dr. Physick in removing a tumor from the face of a young man who fainted. During the period of syncope, Dr. Physick observed how differently persons bore pain. Whilst once performing an operation of an unusually painful nature upon a man, and wondering at his not crying out with agony, he was surprised at hearing him calmly observe, "Dr. Physick, how very keen your knives are. It is really a pleasure to be cut with them."

The second case occurred under Dr. E.'s own observation, in the person of a near relative, a young lady, who, whilst very young, would never suffer any thing to be done to her teeth. All kinds of entreaties and bribes were exhausted upon her, and the result was, that her teeth, being too much crowded, began to interfere with her pretty looks. As soon as she attained an age when these became more fully appreciated, she cheerfully consented to visit a dentist, who found it necessary to extract no less than five teeth, all strongly rooted. Being her attendant, he was extremely surprised by such an exhibition of fortitude in one whom he knew to be endowed with exquisite sensibilities; and asking her immediately after the operation, if she had not suffered intensely? was still more surprised to hear her say, that the only pain she had experienced was from the apprehension of the suffering he might have on her account. If mesmeric processes had been resorted to, this of course would have been considered a conclusive demonstration of their efficac y.

Dr. Emerson observed, that this boon, for such hethought this natural resource might appropriately be called, was most frequently possessed by persons endowed with exquisite sensibility, against the effects of which it might be regarded as a protection. Where the suspension of feeling was induced by mesmeric manipulation, he considered the phenomenon as altogether the result of that extraordinary power which the mind, when deeply impressed by a sense of mystery, or some other obscure agency, could exert over the nervous system, and both with such agencies and phenomena, it would be well for the profession to become better acquainted.

Dr. Evans inquired of Dr. Parrish whether in the course of his investigation into the effects of the inhalation of ether or chloroform, he had met with or heard of any case of deranged mental manifestations resulting therefrom? He had been informed of three cases said to have been produced by etherization, but had had no opportunity of verifying the statement, and he would feel much indebted to any of his professional brethren who had seen such effect resulting from the use of either of the anæsthetic agents, who would communicate it to him, or allow him the opportunity of observing it.

In regard to what had just been said by Dr. Emerson in relation to some peculiar power in the human system to suppress the feeling of pain, he thought that whenever insensibility to pain, under causes usually producing it, existed, it must be the result of disease in the nerves or centre of sensation; and he had repeatedly witnessed cases of this kind. In two instances of men deprived of the use of their reason, who had come under his notice, the sense of feeling was so completely destroyed, that they respectively stood near enough to a hot stove to burn the glutei muscle so deeply that large sloughs came out, and yet were not aware of it at the time. They appeared otherwise to enjoy good health. And at the present time he had a lady under his care, who, in the early stages of her disease, which appeared to be spinal, was so completely deprived of feeling that a pin could be thrust into her flesh almost anywhere, without her being conscious of it. It was upon this sensitive portion of the nervous mass that the anæsthetic agents acted. From theorising on the action of these agents he had been decidedly opposed to their use; but having been invited by Dr. Horner to be present at his operations before the class at the University, in 1850–51, he had seen them used there very freely, and with the happiest results, so that he had been induced to alter his opinion, and he considered their employment justifiable, where extensive operations, usually giving rise to great pain, were to be performed.

Dr. Wiltbank said, that as he had been among the objectors to the use of anæsthetics in midwifery, he felt it incumbent on him to make a few remarks. Until within the last year he had opposed these agents on theoretic grounds. He feared that they would subject the parturient female to danger from lacerations, hemorrhages, convulsions, fevers, and many other evils. He had argued himself into the belief that they must, almost necessarily, injure both mother and child. The accumulating testimony in their favor, however, and the urgent solicitations of his patients, induced him to give them a cautious trial; and he must say that his opinions had undergone a very material change. He found that he had exaggerated the objections and undervalued the advantages to be derived from them; but he could not endorse the opinions of those who advocated their employment in all, or even a majority of labors. Like every other article in the Materia Medica, anæsthetic agents should be restricted to the proper cases. Their *great* use, he thinks, is to allay excessive, irregular and inefficient pains, to promote sleep and to prevent exhaustion. To illustrate his meaning, he cited two interesting cases. The first was that of a lady he saw in consultation, who had been in active labor sixty hours. The membranes were ruptured, the head large and firmly ossified was immovably fixed in the superior strait, and although the pains were frequent and severe, no advance was made. Her general system was dangerously suffering from the long continuance of the pains and the loss of sleep. Ether here acted like a charm. It allayed pain, secured sleep, and refreshed and invigorated the patient. The use of the anæsthetic agent was kept up, with occasional intermissions, for thirty-six hours, and he thinks that without it, her life must have been inevitably lost; she recovered well. A second case, still more strikingly illustrative of the benefits of anæsthetics, properly applied, fell under his notice. This was a lady, who, when near the close of gestation, being exposed to cold in attending upon a sick child, was seized with severe and continuous pains. The whole abdomen was hard, the body of the womb firmly contracted, and the pains violent and without any distinct intermission. The os uteri, however, was unaffected, and there were no signs of labor. She was bled, and took opiates and antispasmodics in large and repeated doses without any relief. The pains increased until the patient was almost frantic with suffering, and still there were no signs of labor. Chloroform was given with delightful effect. The pains were instantly arrested, the irregular contractions were subdued, and the patient slept sweetly. In fifteen minutes she awoke with slight bearing pain; the os was now found to be dilating, and in one hour and a half the delivery was safely and easily accomplished.

